# Right ventricular ejection fraction in postoperative cardiac surgery patients is independently associated with ICU morbidity and mortality

**DOI:** 10.1186/2197-425X-3-S1-A112

**Published:** 2015-10-01

**Authors:** IT Bootsma, F de Lange, M Koopmans, J Haenen, PW Boonstra, EC Boerma

**Affiliations:** Intensive Care Medicine, Medical Centre Leeuwarden, Leeuwarden, Netherlands; Cardiothoracic Anesthesiology, Medical Centre Leeuwarden, Leeuwarden, Netherlands; Cardiothoracic Surgery, Medical Centre Leeuwarden, Leeuwarden, Netherlands

## Introduction

Left ventricular heart failure is a well-known risk factor in cardiac surgery. However, data on the clinical relevance of right ventricular (RV) failure are limited.^1^

## Objectives

To establish the prognostic implications of RV failure in a large series of post cardiac surgery patients.

## Methods

We performed a single-centre retrospective analysis of all high risk cardiac surgery patients in a four year period. By protocol these patients were equipped with a pulmonary artery catheter (Vigilance^®^, Baxter), enabling continuous RV ejection fraction (RVEF) measurements. RVEF was categorized into three subgroups: RVEF < 20%, 20-30% and >30%. Demographic data and hemodynamic variables were recorded. Primary outcome was predefined as the correlation between the average RVEF over the first 24 hours in the ICU and markers of morbidity.

## Results

A total of 1115 patients were included. Patient characteristics are summarized in table 1. Patients with an RVEF < 20% had a significant longer duration of mechanical ventilation and lengths of stay in the ICU, higher ICU mortality, and increased use of inotropes and fluids. In a multivariate logistic regression model, RVEF appeared to be an independent risk factor for duration of mechanical ventilation, length of stay ICU, and ICU mortality.

**Figure 1:**
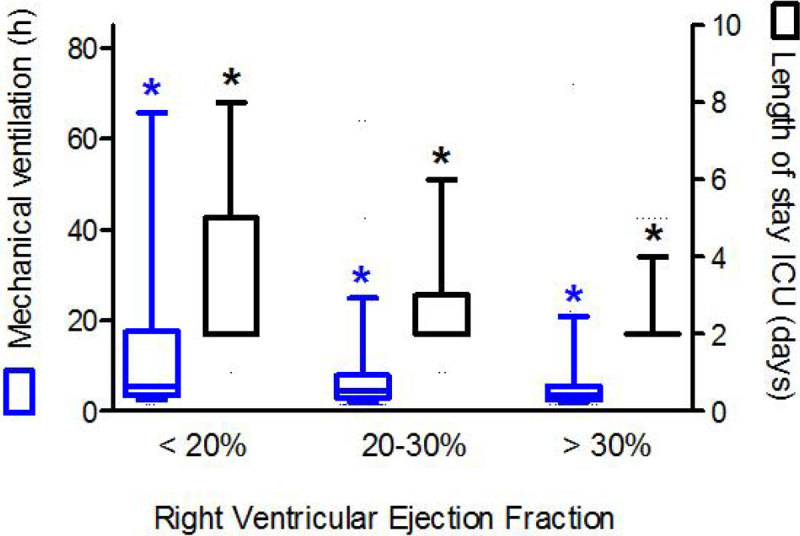
Figure 1

**Table 1:** Baseline characterstics.

	RVEF <20% (N = 218)	RVEF 20-30% (N = 750)	RVEF >30% (N = 147)	P-value
Age (years)	74[67-79]	70[63-77]	65[58-73]	< 0.001
Diabetes (%)	18	21	16	0.40
COPD (%)	22	17	12	0.04
NYHA III or IV (%)	50	37	35	0.01
Poor LVEF (%)	21	12	7	< 0.001
Additive euroSCORE 1	8[6-10]	7[5-9]	6[4-8]	< 0.001
Aortic cross-clamp (min)	98[70-128]	97[68-139]	93[65-134]	0.68
CABG (%)	13	12	11	0.43

Data are median [interquartile range] or percentage. RVEF right ventricular ejection fraction. COPD chronic obstructive pulmonary disease. euroSCORE European system for cardiac operative risk. NYHA New York heart association. LVEF left ventricular ejection fraction. CABG coronary-artery bypass grafting.

**Table 2:** Outcome.

	RVEF <20% (N = 218)	RVEF 20-30% (N = 750)	RVEF > 30% (N = 147)	P-value
Mechanical ventilation (hours)	5,5[3,5-17,6]_a_	4,5[3,0-8,0]_b_	3,5[2,5-5,5]_c_	< 0,001
Length of stay ICU	2[2-5]_a_	2[2-3]_b_	2[2-2]_c_	< 0,001
Survival ICU (%)	96_a_	99_b_	99_b_	0,01
Use of inotropic drugs (%)	75_a_	61_b_	48_c_	< 0,001
Fluid balance (litres)	1,9[1,2-3,2]_a_	1,6[0,8-2,8]_b_	1,1[0,3-2,0]_c_	< 0,001

Data are median [interquartile range] or percentage. RVEF right ventricular ejection fraction. ICU intensive care unit. Groups are significantly different when p < 0,05. Subscript letters: Different letters indicate a significant difference of p < 0,05 between groups.

## Conclusions

A RVEF < 20% is independently associated with increased ICU mortality and morbidity in high risk postoperative cardiac surgery patients.
